# Laboratory Findings and Clinical Features in IgA Vasculitis: Identifying Predictors of Kidney Involvement and Disease Relapse in Pediatric Patients

**DOI:** 10.3390/jcm14093055

**Published:** 2025-04-29

**Authors:** Zofia Podraza, Karol Poplicha, Tomasz Ufniarski, Jarosław Ucieklak, Natalia Łysiak, Małgorzata Mizerska-Wasiak

**Affiliations:** 1 Science Students’ Association at Department of Pediatrics and Nephrology, Medical University of Warsaw, 02-091 Warsaw, Poland; zosia.podraza@gmail.com (Z.P.); karol41299@gmail.com (K.P.); tomaszeku@gmail.com (T.U.); ucieklakj@gmail.com (J.U.); natalysiak123@gmail.com (N.Ł.); 2 Department of Pediatrics and Nephrology, Medical University of Warsaw, 02-091 Warsaw, Poland

**Keywords:** IgA vasculitis, IgA vasculitis nephritis, pediatric nephrology, disease recurrence

## Abstract

**Objective:** This study aimed to identify clinical and laboratory predictors of kidney involvement and disease relapse in pediatric patients with IgA vasculitis (Immunoglobulin A vasculitis, IgAV). **Materials and Methods:** A retrospective cohort study was conducted on 173 children diagnosed with IgAV at the Children’s Clinical Hospital of the Medical University of Warsaw between 2018 and 2022. Patients were categorized into groups based on renal involvement (IgAVN+ vs. IgAVN−) and disease recurrence. The analysis included demographic data, clinical manifestations, allergy history, presence of infection, duration of hospitalization, relapse occurrence, the interval between the first and second hospitalization, and laboratory markers. **Results:** Renal involvement was observed in 42% of cases, while disease recurrence occurred in 9.25% of patients. IgAVN+ patients were older, had longer hospital stays, and more frequently exhibited gastrointestinal symptoms, consistent with previous research. A history of allergic conditions was more prevalent in both the IgAVN+ and recurrence groups. An increase in IgA levels over time was associated with a higher risk of nephropathic development. Patients with recurrences had higher IgM levels and an elevated neutrophil-to-lymphocyte ratio (NLR) (*p* = 0.07). In the ROC (Receiver Operating Characteristic) analysis, a cutoff value of 1.67 for NLR (AUC 0.71; *p* = 0.0002; sensitivity 0.87; specificity 0.58) was identified as a risk factor for disease recurrence. **Conclusions:** Older age at disease onset, gastrointestinal involvement, and allergies are associated with renal involvement in pediatric IgAV. Immune dysregulation, reflected by elevated NLR and IgM, may contribute to disease recurrence. It is important to monitor changes in IgA levels over time, as an increase in IgA concentration is a risk factor for the development of nephropathy. Additionally, calculating the NLR is recommended, as it may indicate the probability of disease recurrence.

## 1. Introduction

IgA vasculitis (Immunoglobulin A vasculitis, IgAV) is a type of systemic small-vessel vasculitis characterized by leukocytoclastic inflammation with neutrophilic infiltration, accompanied by immune complex deposits predominantly containing IgA1. It is the most common form of vasculitis in children, with an annual incidence of 3–27 cases per 100,000 [1]. Approximately 90% of cases occur in children aged 2–10 years, with the peak incidence observed between the ages of 4 and 7 years [2]. A preceding history of upper respiratory tract infection or exposure to specific antigens from foods, insects, medications, or vaccines is frequently observed before the onset of IgAV, suggesting that infections or mucosal antigen exposure may trigger the disease’s pathogenesis [3].

The disease typically follows a self-limiting course, with an average duration of approximately four weeks [4]. The characteristic initial manifestation is purpura, although other organ systems, including the joints, gastrointestinal tract, and kidneys, may also be involved. [5]. Renal involvement in IgAV manifests as urinary abnormalities, such as hematuria with/or without proteinuria. It may progress to more severe conditions such as nephritic syndrome, nephrotic syndrome, or chronic kidney failure. In most cases, IgAV nephritis (IgAVN) is mild and primarily characterized by isolated urinary abnormalities without other symptoms. Chronic kidney disease has been observed in 1–15% of pediatric IgAVN cases [6]. In the acute phase of IgAV, life-threatening complications such as severe gastrointestinal bleeding, intestinal intussusception, or bowel perforation may occur, requiring increased clinical vigilance [7].

Disease relapse is relatively uncommon, and its underlying mechanisms remain poorly understood [8]. Recurrent IgAV is commonly defined as the reappearance of purpura and other characteristic symptoms of IgAV after complete remission [9]. Recurrences are reported in approximately one-third of cases, typically occurring within four months of the resolution of initial symptoms [10,11].

Renal involvement is a crucial determinant of disease relapse. Recurrent IgAVN may progress to end-stage renal disease, highlighting the need to identify risk factors associated with relapse and renal involvement.

This study aims to identify predictors of recurrence and renal involvement in affected patients. A better understanding of these risk factors is essential for preventing disease progression and improving long-term outcomes.

## 2. Materials and Methods

### 2.1. Population and Study Design

This retrospective cohort study was conducted at the Children’s Clinical Hospital of the Medical University of Warsaw between January 2018 and January 2022. Data were collected from electronic medical records, using the International Statistical Classification of Diseases and Related Health Problems (ICD-10) M31.8 and D69 codes. Patients were diagnosed with IgAV based on the 2010 EULAR/PRINTO/PRES criteria. The inclusion criteria encompassed children aged 0 to 18 who required at least one hospitalization for IgAV. Demographic data, clinical features, and laboratory findings were extracted from the CGM Clininet IT System (CompuGroup, Koblenz, Germany).

### 2.2. Data Collection

For each patient, the following information was documented: sex and age at disease onset; clinical manifestations involving the skin, gastrointestinal tract, joints, and kidneys; the presence of food, respiratory, and skin allergies; and information on the presence of infection, duration of hospitalization, occurrence of relapses, and the interval between the first and second hospitalization.

Skin manifestations were defined as the occurrence of purpura, ranging from isolated lesions to extensive skin involvement. Gastrointestinal involvement was characterized by abdominal pain, vomiting, diarrhea, and the detection of occult blood in stool. Articular involvement included joint pain or swelling. IgAVN was diagnosed based on kidney biopsy findings following Kidney Disease: Improving Global Outcomes (KDIGO) guidelines.

Laboratory tests included white blood cell (WBC) count, lymphocyte and neutrophil percentages, hemoglobin (HGB) levels, and platelet (PLT) count. The neutrophil-to-lymphocyte ratio (NLR) was calculated from these results. Additionally, serum levels of urea, creatinine, immunoglobulins G, A, M (IgG, IgA, IgM), and complement components C3 and C4 were collected. The estimated glomerular filtration rate (eGFR) was calculated using the Schwartz formula, with a value >90 mL/min/1.73 m^2^ considered normal in patients older than one year. Age-dependent reference ranges were applied for both WBC and hemoglobin. PLT counts were determined using various methods, including impedance with hydrodynamic focusing, optical analysis, or fluorescence techniques (Sysmex, Japan), with a reference range of 150–450 × 10^3^/µL. Serum creatinine and urea concentrations were evaluated using age-dependent reference ranges. Immunoglobulins (IgA, IgG, IgM) and complement components (C3 and C4) were measured using immunonephelometric methods (Siemens, Germany). The reference ranges for immunoglobulins varied by age, whereas reference ranges for complement components were 90–180 mg/dL for C3 and 10–40 mg/dL for C4.

Patients without renal involvement received standard therapy, while the treatment strategy for those with nephropathy was individualized based on kidney biopsy findings and clinical presentation. Therapeutic interventions were not systematically analyzed as part of this study.

### 2.3. Statistical Analysis

The data were analyzed using the Shapiro–Wilk test to assess the normality of distribution. For normal distributed variables, the mean and standard deviation (SD) were calculated, and Levene’s test was applied to evaluate the homogeneity of variance. The Student’s *t*-test was used for independent or paired samples as appropriate. For non-normally distributed variables, the Mann–Whitney U test was used for independent samples, while the Wilcoxon signed-rank test was applied for paired samples. The Z-test for two independent proportions was utilized to compare the frequency of a given characteristic between groups. A *p*-value of <0.05 was considered statistically significant. The ROC (Receiver Operating Characteristic) analysis was performed and a cutoff value for NLR was identified as a risk factor for disease recurrence. All analyses were performed using Statistica v.13 software (TIBO Software Inc., San Ramon, CA, USA).

## 3. Results

### 3.1. Baseline Characteristics

Table 1 presents the baseline characteristics at the onset of the disease in patients with IgAV. A total of 173 children were enrolled in this study. The male-to-female ratio was 1.08:1, with a mean age of 6.55 years (range: 1.24–17.65 years). Skin involvement was observed in 100% of children, while gastrointestinal symptoms were noted in 42.2%, joint symptoms in 68.2%, and renal involvement in 42.2%. The mean length of hospitalization was 7.72 days (range: 1–64 days).

### 3.2. Comparison of IgAVN− and IgAVN+ Groups

**Figure 1:**
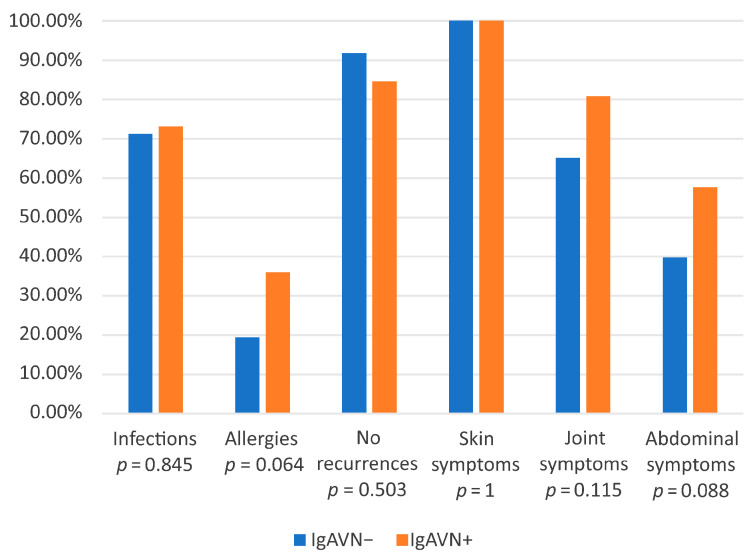
Comparison of the prevalence rates of given clinical features, allergies, infection, and recurrence in the IgAVN− and IgAVN+ groups during the first episode.

Patients were categorized into two groups: those with kidney involvement (IgAVN+) and those without kidney involvement (IgAVN−). Laboratory test results and the prevalence of specific characteristics were compared between these groups during the first and second disease episodes (Table 2 and Figure 1).

**a.**  **First Episode**

During the first episode of the disease, statistically significant differences were observed in patient age (higher in the IgAVN+ group). The mean hospitalization length was significantly longer in the IgAVN+ group compared to the IgAVN− group (20.04 vs. 5.52 days, *p* < 0.05). Abdominal symptoms were more frequently observed in the IgAVN+ group, affecting 57.7% of patients compared to 39.7% in the IgAVN− group (*p* = 0.088). A history of allergies was also more prevalent in the IgAVN+ group, reported in 36% of patients (food allergies: 16.7%, airborne allergies: 12.5%, skin allergies: 8.3%), compared to 19.4% in the IgAVN− group (*p* = 0.064). The frequency of infections preceding the onset of IgAV was similar in both groups: 71.2% in the IgAVN− group and 73.1% in the IgAVN+ group.

**b.**  **Second Episode**

**Figure 2:**
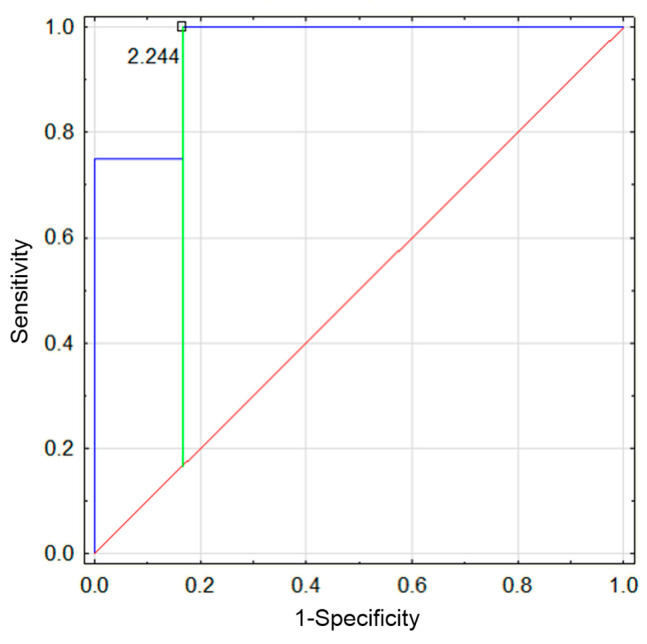
ROC analysis: sensitivity and specificity of the NLR value during the second episode of IgAV for predicting the presence of nephropathy.

During the second episode, hospitalization duration remained significantly longer in the IgAVN+ group compared to the IgAVN− group, with median lengths of stay of 11.5 days versus 4 days, respectively (*p* < 0.05). The NLR was significantly higher in the IgAVN+ group (5.18 vs. 1.4, *p* < 0.05). A ROC (Receiver Operating Characteristic) analysis was performed to establish the cutoff value for NLR beyond which the probability of renal involvement increases. The analysis demonstrated a significantly higher probability of renal involvement above a threshold of 2.24 (AUC 0.96, *p* < 0.05, sensitivity 0.75, specificity 1) (Figure 2).

A higher percentage of IgAVN− patients experienced only one disease episode (91.8%) compared to IgAVN+ patients (84.6%). The interval between the first and second hospitalization was shorter in the IgAVN+ group (103 days) compared to the IgAVN− group (122 days). However, neither of these differences reached statistical significance.

### 3.3. Comparison of Recurrence Group and Group with One Episode

**Figure 3:**
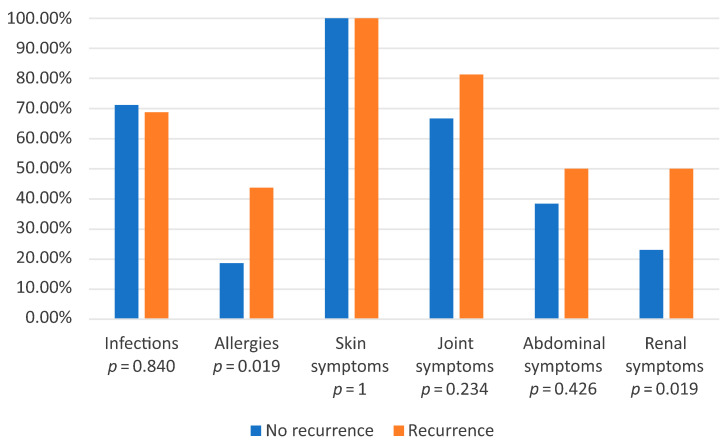
Comparison of the prevalence of given clinical features, infections, and allergies in the recurrence and non-recurrence groups during the first episode.

Patients were also divided into two different groups: those with at least one relapse (defined as the recurrence of purpura or systemic symptoms after clinical resolution) and those without relapse (Table 3 and Figure 3). The analysis focused on data from the first episode to identify potential risk factors for disease recurrence.

**Figure 4:**
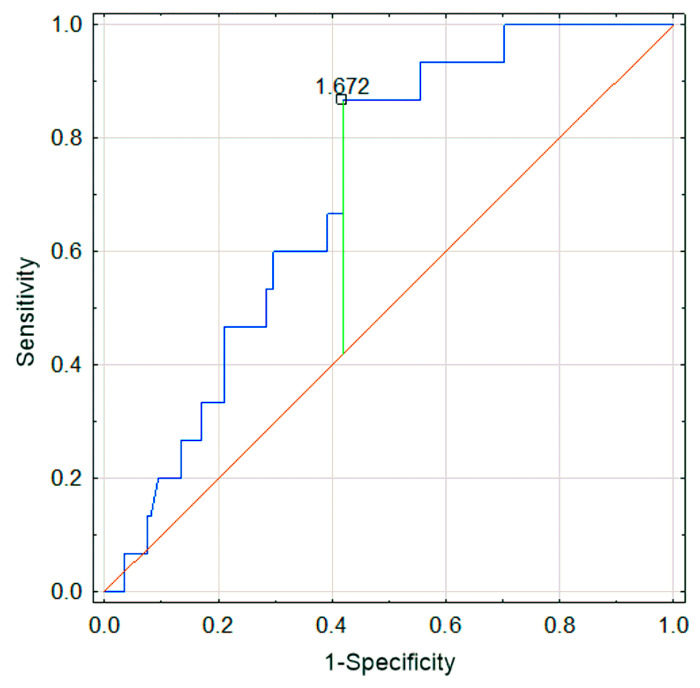
Sensitivity and specificity of NLR during the first episode of IgAV for predicting relapse.

The mean IgM level was significantly higher in the relapse group (147.3 mg/dL vs. 108.9 mg/dL, *p* = 0.022). IgA levels were similar in both groups. The mean age was higher in the relapse group (8.14 years), compared to the non-relapse group (6.38 years, *p* = 0.061). The NLR was also higher in the relapse group (2.4 vs. 1.8, *p* = 0.078). Although these differences did not reach statistical significance, they were close to significance, potentially due to the limited sample size. In the ROC analysis, a cutoff point for NLR was established at 1.67 for the relapse and non-relapse groups (AUC 0.71; *p* = 0.0002; sensitivity 0.87; specificity 0.58) (Figure 4). Values above this threshold were associated with an increased probability of relapse. Additionally, in the recurrence group, a history of allergy was significantly more frequent (*p* < 0.05), and more patients exhibited renal symptoms (50% in the relapse group vs. 23.08% in the non-relapse group, *p* < 0.05).

### 3.4. Comparison of Results Between the First and Second Episodes

When comparing laboratory results between the first and second episodes for all patients, significantly higher levels of IgA and IgM were observed during the second episode (*p* = 0.034 and *p* = 0.042, respectively). Logistic regression analysis was performed, showing that an increase in IgA levels between episodes was associated with an elevated risk of renal involvement (*p* < 0.05).

## 4. Discussion

In this study, during the first episode of IgAV, skin involvement was observed in 100% of cases, joint symptoms in 68.2%, gastrointestinal symptoms in 42.2%, and renal involvement in 42% of cases. Similarly, in a study by Bhimma et al., skin lesions occurred in all cases, while kidney, gastrointestinal, and joint involvement were observed in 40–50%, 50%, and 74% of patients, respectively [12].

Renal involvement remains a major concern in IgAV, as it can lead to long-term complications. Previous studies by Liao et al. and Xiong et al. have demonstrated that the risks of nephropathy and recurrence increase with age at disease onset [13,14]. The present study’s findings support this observation, as the mean age of IgAVN+ patients was significantly higher than in those without renal involvement. Specifically, the overall mean age of patients was 8.26 years in the IgAVN+ group, compared to 6.24 years in the IgAVN− group (*p* < 0.05). Additionally, higher mean age was observed in the recurrence group compared to the non-recurrence group (8.14 years vs. 6.38 years; *p* = 0.061).

Several studies have also highlighted the association between gastrointestinal involvement and the increased risk of IgAVN. A study by Sestan et al. demonstrated that the risk of developing IgAVN, as well as the risk of recurrence, is higher in cases with severe abdominal involvement [15]. Additionally, a study by Mizerska-Wasiak et al. also reported that IgAVN was more frequently observed in patients with abdominal symptoms [16]. Our findings support these observations, as gastrointestinal involvement was more prevalent in the IgAVN+ group (57.7% vs. 39.7%; *p* = 0.088), highlighting the need for the close monitoring of patients with abdominal symptoms for potential kidney involvement. Due to the small sample size, the *p*-value did not reach statistical significance; however, a trend toward significance (*p* < 0.05) was observed. Given this tendency, and the support of our findings in the existing literature, we consider these results to be clinically relevant.

While the etiology of IgAV remains multifactorial, infections have been recognized as a potential trigger [17]. In this study, an infection preceded the onset of symptoms in 70.52% of cases. However, no statistically significant difference was observed between the IgAVN− and IgAVN+ groups, suggesting that infections, although commonly preceding disease onset, are not a determining factor for renal involvement or recurrence in IgAV.

In addition to infections, allergic predisposition has been suggested as a potential contributing factor in IgAV. In our cohort, 20.8% of children had a history of allergies, with higher prevalences in the IgAVN+ group and the recurrence group. Similarly, in a study by Xiong et al., the incidences of renal involvement and recurrent rash were significantly higher in pediatric patients with allergic rhinitis or chronic rhinosinusitis [14]. Furthermore, a study by Ma et al. suggested that exposure to food allergens may act as a triggering factor for IgAV [18]. These findings indicate that allergic conditions may influence disease severity, highlighting the importance of conducting allergy diagnostics to identify potential allergens.

In our study, IgA levels during the first disease episode were significantly higher in the IgAVN+ group, but did not differ between the recurrence and non-recurrence group. Similar associations were observed in a study by Tang et al., where IgA levels varied depending on the presence of nephropathy [19]. However, other studies have not confirmed a relationship between elevated IgA levels and either renal involvement or disease severity in children, highlighting the need for a cautious interpretation of this parameter [20].

Notably, in our study, an increase in IgA levels over time was more frequently observed in the IgAVN+ group, highlighting its potential significance in assessing renal involvement. Therefore, serial IgA measurements and the observation of increasing IgA titers over time may provide valuable prognostic information, facilitating the early identification of patients at higher risk for nephropathy and allowing for timely intervention and optimization of treatment.

Finally, immune dysregulation appears to play a critical role in disease recurrence and severity. NLR, a cellular immune response activation marker, is a crucial indicator of stress and systemic inflammation. It is widely utilized in various medical fields as a reliable and easily accessible biomarker of immune response to both infectious and non-infectious stimuli [21]. In our study, elevated IgM levels and increased NLR in the recurrence group during the first episode, along with the higher NLR in the IgAVN+ group during the second episode, suggest a higher degree of immune system dysregulation during relapses.

Due to age-dependent reference ranges for neutrophil and lymphocyte counts, the NLR also varies with age, and standardized reference values are not well established in the pediatric population. In our study, the mean NLRs were significantly higher than the mean levels reported by Moosmann et al. in a cohort of 60,685 healthy children [22]. However, our analysis did not refer to established reference values; instead, NLR was compared between the IgAVN− and IgAVN+ groups, as well as between patients with and without disease recurrence.

An article by Jaszczura demonstrated that an elevated NLR is associated with a higher risk of organ involvement and a more severe disease course in patients with IgAV [23]. Similarly, Li et al. showed that NLR may serve as a risk marker for renal involvement in adult patients [24]. In the present study, an NLR value above 1.64 was associated with an increased probability of relapse. Therefore, we recommend calculating NLR at diagnosis and closely monitoring patients with NLR > 1.64 for potential relapse. Furthermore, in patients experiencing recurrent episodes, NLR should be reassessed during relapses. An NLR value higher than 2.24 during the second episode was linked to a higher probability of renal involvement, highlighting the importance of regular urinalysis and renal function monitoring in these patients. These findings underscore the prognostic value of NLR in the management of IgAV and support its use as a simple and accessible laboratory parameter in clinical practice. Regular assessment of NLR may help identify patients at increased risk of complications and contribute to more personalized follow-up strategies.

## 5. Conclusions

This study highlights the importance of factors associated with the recurrence and severity of IgAV, particularly renal involvement.

Factors identified in the current study include age, significantly higher in patients with nephropathy; abdominal symptoms, more often associated with the onset of nephropathy (an outlined trend); and a history of allergic disease, significantly more common in the IgAVN+ and relapse groups.

This study also showed that an increase in IgA levels during follow-up may be a risk factor for the development of nephropathy, indicating the potential clinical importance of monitoring IgA dynamics.

No significant associations of infection with renal involvement or disease recurrence were observed.

A novel result of the present study is the determination of the cutoff NLR index as an easily accessible marker of possible disease recurrence. Calculating the NLR is recommended in every patient with IgAV.

Further studies in this group of patients are needed to better understand the mechanisms underlying immune dysregulation.

## Figures and Tables

**Table 1:** Baseline characteristics of the studied patients.

Characteristics	All Patients (N = 173)
**Demographics**	
Male/female	1.08:1
Age (years)	6.55 [1.24–17.65]
**Clinical features**	
Skin involvement (n%)	173 (100%)
Abdominal involvement (n%)	73 (42.20%)
Joint involvement (n%)	118 (68.20%)
Renal involvement (n%)	73 (42.20%)
**Allergies (n%)**	37 (21.39%)
Food (n%)	20 (11.56%)
Respiratory (n%)	13 (7.51%)
Skin (n%)	8 (4.06%)
**Presence of infection (n%)**	122 (70.52%)
**Duration of hospitalization**	7.72 [1–64]
**Occurrence of relapses**	9.25%
**Laboratory Values**	
WBC (10^3^/µL)	10.57 ± 3.91
LIMF (%)	34.98 ± 11.42
NEU (%)	53.41 ± 13.12
HGB (g/dL)	12.39 ± 1.13
PLT (10^3^/µL)	372.1 ± 116.3
Urea (mg/dL)	27.42 ± 7.98
Creatine (mg/dL)	0.40 ± 0.15
IgG (mg/dL)	1060.5 ± 331.4
IgA (mg/dL)	194.5 ± 103.1
IgM (mg/dL)	113.98 ± 56.9
C3 (mg/dL)	113.62 ± 25.1
C4 (mg/dL)	24.7 ± 11.8

WBC—white blood cells; LIMF—lymphocytes; NEU—neutrophils; HGB—hemoglobin; PLT—platelets; IgG—immunoglobulin G; IgA—immunoglobulin A; IgM—immunoglobulin M; C3—complement C3; C4—complement C4.

**Table 2:** Comparison of mean results between the IgAVN− and IgAVN+ groups during the first episode and the second.

	Episode I			Episode II		
FACTORS	IgAVN− (N = 147)	IgAVN+ (N = 26)	*p* *	IgAVN− (N = 12)	IgAVN+ (N = 4)	*p* *
Age (years)	6.24 ± 3.42	8.28 ± 4.01	0.007	7.21 ± 4.19	12.32 ± 6.21	0.080
Hospitalization Duration (Days)	5.52 ± 5.4	20.04 ± 11.14	0.000	4 ± 2.3	11.5 ± 10.3	0.026
Interval Between Hospitalizations (Days)	-	-	-	122 ± 188	103 ± 80	0.848
WBC (10^3^/µL)	10.56 ± 3.90	10.66 ± 4.06	0.908	9.25 ± 1.91	10.68 ± 3.93	0.334
LIMF (%)	35.14 ± 11.26	34.13 ± 12.48	0.686	40.78 ± 11.64	19.53 ± 8.88	0.005
NEU (%)	53.31 ± 13.00	53.96 ± 14.04	0.821	49.73 ± 12.30	69.68 ± 11.51	0.013
HGB (g/dL)	12.41 ± 1.03	12.28 ± 1.58	0.574	12.27 ± 0.86	13.53 ± 1.24	0.039
PLT (10^3^/µL)	371.2 ± 119.8	377.2 ± 96.1	0.814	391.9 ± 72.5	304.5 ± 122.2	0.099
NLR	1.83 ± 1.12	2.05 ± 1.48	0.381	1.4 ± 0.72	5.18 ± 4.89	0.015
Urea (mg/dL)	26.45 ± 7.02	31.84 ± 10.46	0.002	24.3 ± 3.09	27.25 ± 7.93	0.318
Creatinine (mg/dL)	0.37 ± 0.12	0.5 ± 0.23	0.000	0.86 ± 1.71	0.6 ± 0.22	0.767
GFR (ml/min/1.73 m^2^)	134.86 ± 33.69	119.4 ± 132.22	0.052	137.48 ± 67.09	103.25 ± 18.78	0.420
IgG (mg/dL)	1074.3 ± 329.1	1021 ± 341.8	0.500	1156.8 ± 474.0	1550	0.460
IgA (mg/dL)	178.7 ± 94.8	252.8 ± 113.1	0.001	244 ± 93.7	342 ± 58	0.199
IgM (mg/dL)	118.7 ± 57.0	98.9 ± 55.0	0.138	162.9 ± 86.1	280	0.241
C3 (mg/dL)	113.9 ± 27.1	112.8 ± 18.3	0.857	-	-	-
C4 (mg/dL)	25.3 ± 13.1	22.9 ± 6.3	0.378	-	-	-

WBC—white blood cells; LIMF—lymphocytes; NEU—neutrophils; HGB—hemoglobin; PLT—platelets; NLR neutrophil-to-lymphocyte ratio; GFR—glomerular filtration rate; IgG—immunoglobulin G; IgA—immunoglobulin A; IgM—immunoglobulin M; C3—complement C3; C4—complement C4; * *p*—*p*-value calculated using Student’s *t*-test.

**Table 3:** Comparison of mean results between the recurrence and non-recurrence groups during the first episode.

FACTOR	Recurrence Group (N = 16)	Non-Recurrence Group (N = 157)	*p* *
Age (years)	8.14 ± 5.06	6.38 ± 3.37	0.061
Interval Between Hospitalizations (Days)	117.2 ± 164.8	-	-
WBC (10^3^/µL)	11.88 ± 3.96	10.44 ± 3.90	0.176
LIMF (%)	28.12 ± 7.20	35.68 ± 11.55	0.014
NEU (%)	60.99 ± 7.77	52.64 ± 13.32	0.018
HGB (g/dL)	12.41 ±1.03	12.28 ± 1.58	0.574
PLT (10^3^/µL)	369.47 ± 61.18	372.36 ± 120.57	0.927
NLR	2.4 ± 0.9	1.8 ± 1.2	0.078
Urea (mg/dL)	26.63 ± 5.74	27.52 ± 8.24	0.683
Creatinine (mg/dL)	0.39 ± 0.15	0.4 ± 0.15	0.888
GFR (ml/min/1.73 m^2^)	143.20 ± 38.00	129.7 ± 33.4	0.258
IgG (mg/dL)	1025.8 ± 394	1066.7 ± 321.6	0.673
IgA (mg/dL)	220.5 ± 110.1	190.9 ± 102.1	0.299
IgM (mg/dL)	147.3 ± 92.9	108.9 ± 48.2	0.022
C3 (mg/dL)	116.2 ± 21.4	113.2 ± 25.7	0.681
C4 (mg/dL)	21.5 ± 4.8	25.2 ± 12.5	0.282

WBC—white blood cells; LIMF—lymphocytes; NEU—neutrophils; HGB—hemoglobin; PLT—platelets; NLR neutrophil-to-lymphocyte ratio; GFR—glomerular filtration rate; IgG—immunoglobulin G; IgA—immunoglobulin A; IgM—immunoglobulin M; C3—complement C3; C4—complement C4; * *p*—*p*-value calculated using Student’s *t*-test.

## Data Availability

The data analyzed in this study are available from the corresponding author upon reasonable request.
